# New Approach to Preparation of Heat-Resistant “Lola-M” Fiber

**DOI:** 10.3390/ma12213490

**Published:** 2019-10-25

**Authors:** Igor I. Ponomarev, Ivan Y. Skvortsov, Yulia A. Volkova, Ivan I. Ponomarev, Lydia A. Varfolomeeva, Dmitry Y. Razorenov, Kirill M. Skupov, Mikhail S. Kuzin, Olga A. Serenko

**Affiliations:** 1 A.N. Nesmeyanov Institute of Organoelement Compounds of Russian Academy of Sciences, Vavilova St., 28, Moscow 119991, Russia; 2 A.V. Topchiev Institute of Petrochemical Synthesis of Russian Academy of Sciences, Leninsky Av., 29, Moscow 119991, Russia; amber5@yandex.ru (I.Y.S.);

**Keywords:** wet spinning, polynaphthoylenebenzimidazole, heterocyclic polymer, polymer fibers, coagulant, polymer solution rheology, viscoelasticity, fiber strength, fiber elastic modulus, fiber morphology

## Abstract

A new approach to the synthesis of polynaphthoylenebenzimidazoles and heat resistant fiber spinning has been developed using an environmentally friendly and energy efficient method, which operates with solutions of pre-polymers based on 3,3’,4,4’-tetraaminodiphenyl ether and 1,4,5,8-naphthalenetetracarboxylic acid dianhydride in N-methylpyrrolidone. Rheological properties of polymer reaction solutions and appropriate coagulant mixtures were investigated for further wet spinning process. The coagulation process was investigated through microscopic observation of solution droplets which imitate jet/fiber cross section surrounded with coagulants of different composition. For the case of the most optimal viscoelastic properties of dopes the best coagulant was found to be a ternary mixture ethanol/water/NMP (20/10/70). Fibers were prepared through the wet spinning from pre-polymers of various molecular weight characterized by intrinsic viscosity. As a result, complex yarns were spun, and their morphology was characterized and mechanical properties were measured. The strength of ~300 MPa and elastic modulus of ~2 GPa and elongation at break of ~20% were reached for the best fibers at average diameter of ~20 µm. After heat treatment “Lola-M” fibers do not burn and do not support combustion in open flame.

## 1. Introduction

**Figure 1:**
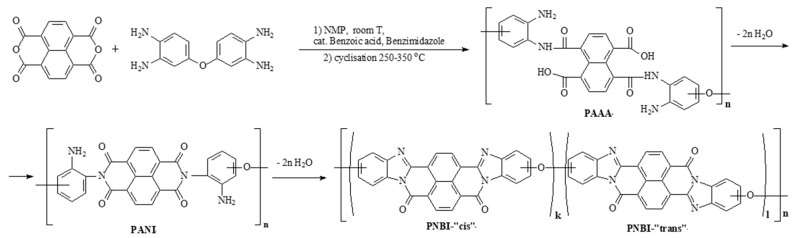
Scheme of PNBI synthesis.

Ladder and semi-ladder polynaphthoylenebenzimidazoles (PNBI) based on aromatic tetraamines and 1,4,5,8-naphthalenetetracarboxylic acid dianhydride have unique thermal, heat- and fire-resistant properties among all organic polymers. Technologies for obtaining prominent fibers known as BBB (polymer based on 1,4,5,8-naphthalenetetracarboxylic acid dianhydride and 3,3’-diaminobenzidine), BBL (polymer based on 1,4,5,8-naphthalenetetracarboxylic acid dianhydride and 1,2,4,5-tetraaminobenzene) and "Lola" from PNBI solutions in sulfuric acid were developed in 1960s and 1970s both in the United States and the Soviet Union [1,2,3,4,5]. “Lola” fiber is based on PNBI polymer synthesized from 1,4,5,8-naphthalenetetracarboxylic acid dianhydride and 3,3’,4,4’-tetraaminodiphenyl ether in polyphosphoric acid (PPA). The obtained from PPA polymer is soluble in 98% sulfuric acid and can be spun through wet spinning process into water-sulfuric acid coagulation bath. Then fibers are subjected to washing and thermal stretching. A modern modification of PNBI fiber (“Lola-Modern” or “Lola-M”) is obtained from a pre-polymer solution in N-methyl-2-pyrrolidone (NMP). PNBI synthesis was usually performed in one-step polycondensation in polyphosphoric acid at 180–200 °C for 8–10 h. That process required special acid-resistant equipment, was not energy-efficient and produced a lot of acidic wastes. Despite these facts, a pilot production of "Lola" fiber reached one ton per year but ceased with collapse of the USSR. In current work the pre-polymers for "Lola-M" fiber production are synthesized in environment friendly and energy-efficient process from 3,3′,4,4′-tetraaminodiphenyl ether and 1,4,5,8-naphthalenetetracarboxylic acid dianhydride in NMP and used for fiber production. Pre-polymer fibers are converted to semi-ladder structure by heat treatment at 250–350 °C as shown in Figure 1.

## 2. Materials and Methods 

### 2.1. Materials

#### 2.1.1. Monomers and Solvents

3,3′,4,4′-tetraaminodiphenyl ether (Rubezhnoe plant, Rubezhnoe, Ukraine) M_p_ 155–156 °C and 1,4,5,8-naphthalenetetracarboxylic acid dianhydride (VNIPIM, Tula, Russia) were dried at 100 °C under vacuum before use. Benzimidazole, benzoic acid and NMP (Thermo Fisher Scientific, Acros Organics, Waltham, MA, USA) were used as received. The following coagulants for wet spinning were tested: water, ethanol and their mixtures with NMP. All these reagents were supplied from Ekos-1 (Moscow, Russia).

#### 2.1.2. Synthesis of PANI

2.3027 g (0.01 mol) of 3,3′,4,4′-tetraaminodiphenyl ether, 2.6818 g (0.01 mol) of 1,4,5,8-naphthalenetetracarboxylic acid dianhydride, 0.24 g (0.00196 mol) of benzoic acid, 0.24 g (0.002 mol) of benzimidazole and 20 mL of anhydrous NMP were placed in the flask and stirred in Ar flow for 24 h at room temperature forming viscous solution of polyaminonaphthoyleneimide (PANI). Reaction mixture was then stirred at 50–60 °C until the agitator was almost stopped due to very high viscosity. After dilution of pre-polymer solutions with NMP they were used for fiber production.

#### 2.1.3. PANI Solutions

Various series of PANI solutions in NMP obtained in two different syntheses were selected for the analyses of (P1–P4). The used solvent was NMP. The solution concentrations and intrinsic viscosities of the polymers are presented in Table 1. The solutions obtained after synthesis were divided into several batches. P1 reaction solution with polymer content of 11.6% mass. was additionally aged to increase molecular weight at 70 °C for 5 h under slow stirring with an anchor stirrer. As a result, the solution P2 was obtained. The following synthetic sample P3 with polymer concentration of 13.0% mass. was kept at 25 °C for 24 h to obtain P4 solution.

### 2.2. Selection of a Coagulant

The selection of an appropriate coagulant plays very important role in fiber spinning because it controls the structure and morphology of the obtained fibers [7,8,9] and films [10]. Morphology evolution of solution droplet surrounded by a coagulant was studied (modeling the jet/fiber cross-section) through microscopic methods. Thickness of the droplet was ~0.1 mm and its average diameter was ~1.5 mm. The observation of polymer solution-coagulant interaction was carried out using the Biomed 6 PO microscope (Biomed, Moscow, Russia). Tests for all coagulants were performed at 25 °C.

### 2.3. Rheology

The rheological properties of solutions were measured at a steady state and oscillation regimes of the shear strain using rotational rheometer Anton Paar MCR 301 (Anton Paar GmbH, Graz, Austria) in temperature range of 25–60 °C. The behavior of “living” solutions was tested using an operating unit of two geometries. Coaxial cylinders with diameter of the inner cylinder of 10 mm and gap of 0.42 mm were used for the prolong kinetics measurements. Cone and plate unit with cone diameter of 25 mm and angle between cone and plate of 2° were used for the tests at different shear rates and temperatures.

The flow curves were recorded at the shear rate range of 10^−2^–10^4^ s^−1^, the amplitude dependencies of the complex elasticity modulus were recorded at the strain range of 0.06–62 rad s^−1^ at constant frequency of 1 and 80 Hz, the frequency dependences of the storage and loss moduli were recorded at the angular frequency range of 0.6–628 rad s^−1^. Kinetics measurements were performed at non-destructing mechanical action in the cylinder-cylinder unit at constant frequency of 1 Hz and strain of 1%. The edge of the gap was covered by silicone oil to prevent gel formation due to solution interactions with wet air. 

The scanning of frequency was performed every 30 min at constant temperature to determine frequency dependences of moduli at the strain of 1%. Kinetics of polymer property change was determined by rheological measurements at constant frequency and deformation (one time in 10 s) and by recording of frequency dependences (every 30 min). The experiments were carried outat 60 °C during 4 h, i.e., until the solution became a gel and was unsuitable for the spinning. Thus change intensity of viscoelastic characteristics at constant temperature was estimated. These data allow to choose the heating time for further conditioning of the dopes. 

The intrinsic viscosities were measured using handmade Ubbelohde viscometer at 25 °C from the flow time for diluted samples. Each concentration was measured at least 10 times. Flow time of solvent was more than 100 s. Flow times for P3 solution for different concentrations are shown in Table S1 in Supplementary Materials.

### 2.4. Fiber Spinning

**Figure 2:**
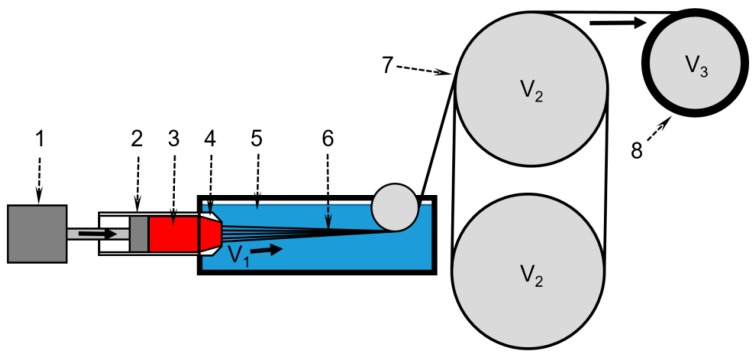
Laboratory stand scheme for wet spinning. 1—engine; 2—100 mL metallic syringe; 3—dope; 4—multifilament spinneret with a die diameter of 80 µm; 5—coagulation bath at 25 °C; 6—solution jets/yarn; 7—winding roller; 8—winding spool. Variable V1 is a linear flow speed from the spinneret and V2 is a winding speed. V2 is equal to V3.

Fibers from PANI solutions were spun on a laboratory stand shown schematically in Figure 2. The main attention was paid to a producing of homogeneous defect-free fibers with sufficient mechanical strength.

Spinning was carried out at a constant flow rate of the solution through a multi-filament cap-like spinneret with a hole diameter of 80 µm and a total number of holes of 100. The winding speed was 1 m min^−1^, the spinbond hood was estimated as V_2_/V_1_~1.5. V_2_ was equal V_3_. The spinning process was performed at 25 °C.

### 2.5. Fiber Characterization

The diameter of each fiber was determined as the average of ten measurements at different locations along the fiber. To perform these measurements, the Biomed 6PO optical microscope coupled with a Touptek XFCAM1080PHD camera (Hangzhou ToupTek Photonics Co., Hangzhou, China) with 60× magnification was used. An accuracy of measurements was ±0.3 µm. For every yarn at least 10 filaments were examined. Inhomogeneity was characterized by the difference between the maximum and minimum values of the fiber diameter.

Mechanical properties of PANI fibers were measured using the Instron 1122 tensile machine (Instron, Norwood, MA, USA) at basic filament length of 10 mm and extension rate of 10 mm min^−1^. All measurements were performed at 23 ± 2 °C. The reported results were averaged for at least 10 tests.

## 3. Results and Discussion

### 3.1. Rheological Properties

**Figure 3:**
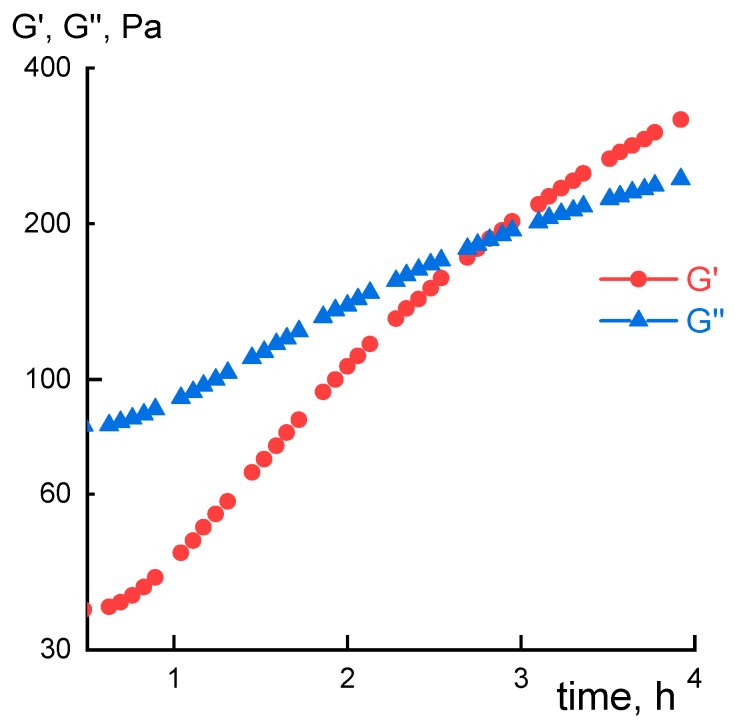
Kinetics of viscoelastic characteristics growth for P3 solution at 60 °C in the domain of linear viscoelasticity (deformation 1%, frequency 1 Hz).

Kinetic of PANI molecular weight increase was monitored by observing changes in solution rheological properties. Evolution of dynamic moduli for P3, taken as an example, is shown on Figure 3. 

**Figure 4:**
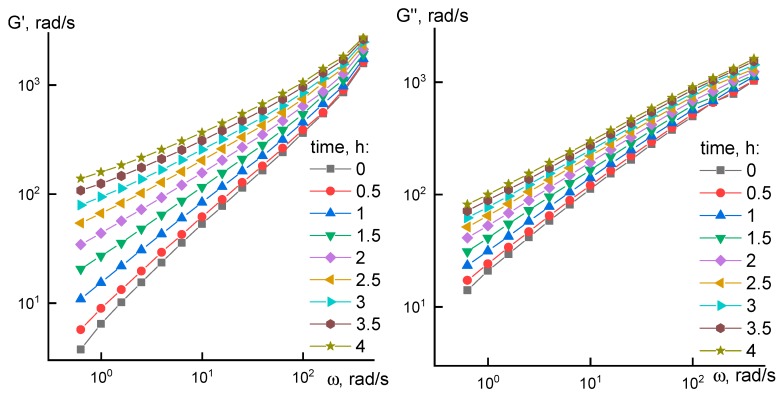
Frequency dependences of viscoelastic properties for P3 sample heated at 60 °C at different times.

During the measurement, a frequency scanning of the viscoelastic characteristics G’ and G’’ was periodically performed to obtain frequency dependences. Frequency dependences for G’ and G’’ at different heating time are shown in Figure 4.

Initially, P3 solution is in a form of viscoelastic structured liquid. At low frequencies the tangent values of frequency dependency slope angles for elastic and loss moduli are equal to 1 and 0.74, subsequently. As time passes significant changes happen in the solution, and exponent signs of frequency dependences gradually decrease. The decrease of elastic modulus growth intensity with time happens faster than the same for loss modulus. Dependency slope reaches ~0.55 in the end zone. Further heating at 60 °C leads to slow gel formation. Unlike concentrated cellulose solutions where curve slopes gradually decrease with a polymer concentration growth [11] over the entire frequency range, in our case, the tendency to gel formation is observed mostly at low frequencies. It might be explained by possible weak physical net formation which may disintegrate at higher frequencies.

**Figure 5:**
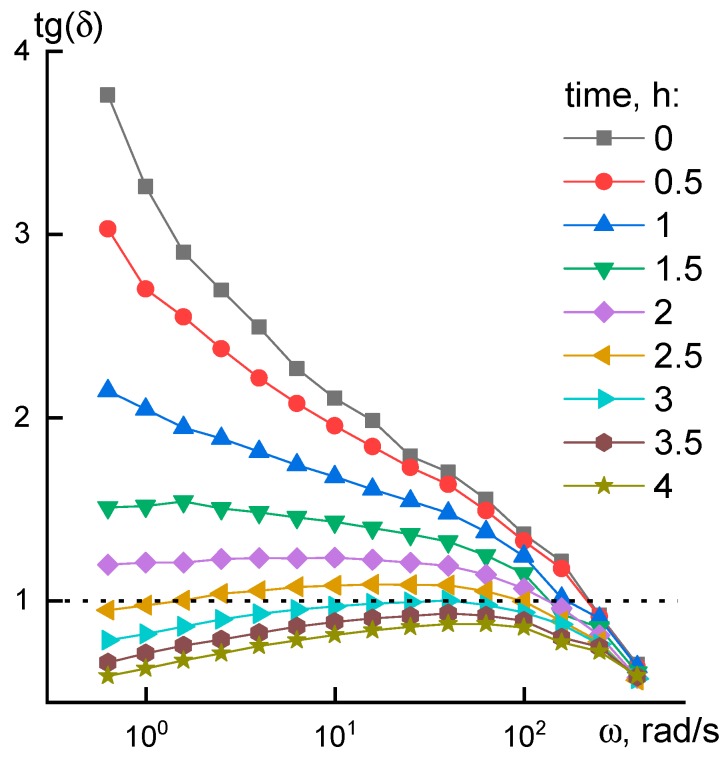
Frequency dependences of the loss tangent for P2 solution in time at 60 °C.

Evolution in rheological properties of solutions is clearly seen from the frequency dependency of tangent of phase shift angle (Figure 5), which is equal to G”/G’. 

In cases when viscoelastic liquid G” > G’ and tan δ > 1 the situation for elastoviscous system is reversed, and tan δ becomes less than 1 (marked with dotted line). Figure 5 shows the process of viscoelastic system to gel system transition at low frequencies after 2.5 h of heating. Analogous transition at high frequencies is due to achievement of a rubber-like plateau. Similar changes in the system were also observed for P3 solution after 12 h at room temperature leading to the formation of P4 solution.

**Figure 6:**
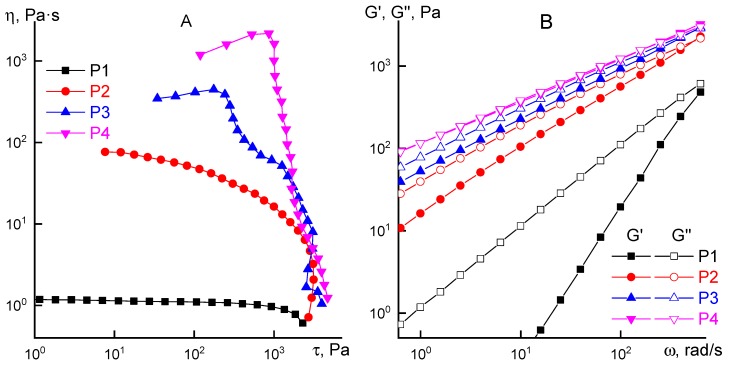
Flow curves (**A**) and frequency dependences of moduli (**B**) for PANI solutions.

Similar experiment was conducted with P1 solution, which is a viscoelastic liquid with frequency dependency slopes of elastic and loss moduli to be 1 and 2 correspondingly. Keeping P1 solution at 70 °C leads to a molecular weight increase and P2 solution formation with higher viscosity, and a presence of a line corresponded to the initial Newtonian viscosity, unlike P3 and P4 solutions. As a result, two groups of solutions with different concentrations, viscoelastic properties and molecular weights of the same polymer were obtained. Flow curves and frequency dependences of the elastic and loss moduli are shown in Figure 6 for all four samples. Additional G’ and G’’ data for P3 and P4 solutions is shown in Figure S1 (Supplementary Materials).

Behavior of the solutions changes significantly with an increase of the polymer molecular weight. Starting from polymer intrinsic viscosity of 1.2 dL g^−1^ (sample P2), it is possible to observe a weak viscosity anomalous behavior. For higher molecular weight polymer P3 and P4 solutions a viscosity anomaly becomes so significant that it would be possible to discuss a yield point, i.e., viscoplastic behavior. One can observe a sharp viscosity decrease in a narrow range of shear stress for such systems. It is probably related to a physical net destruction in a high-molecular structured system with further transition into induced rubber-like state and sample slippage relatively operating unit surfaces. [12,13,14].

**Figure 7:**
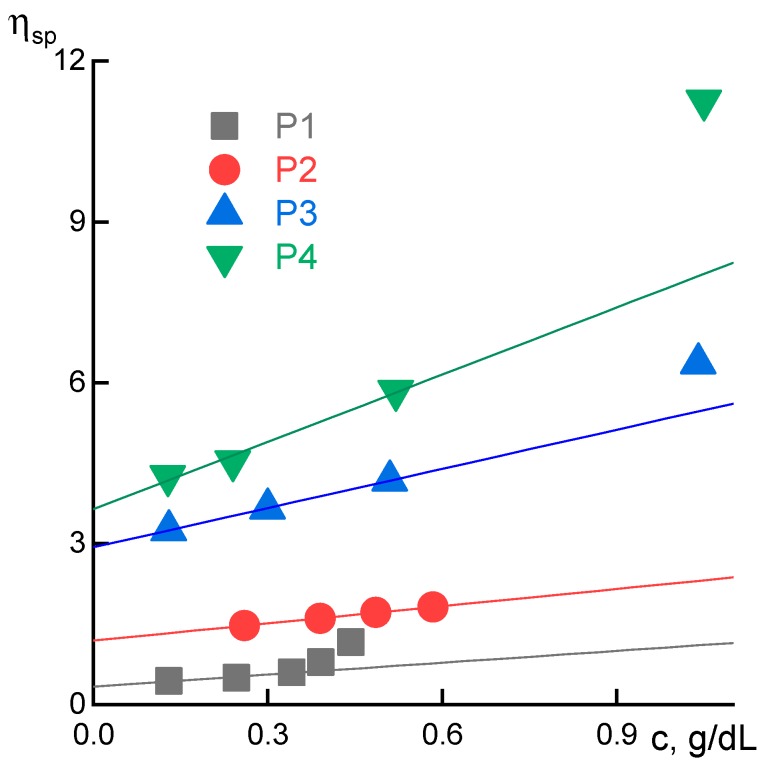
Dependences of the reduced viscosity on concentration for P1–P4 samples at 25 °C.

For quick assessment of the molecular weight change with time, diluted solutions of P1–P4 samples were prepared and intrinsic viscosity was estimated. The reduced viscosity vs. concentration dependences with extrapolation to zero concentration are shown in Figure 7.

For the sample P1 the intrinsic viscosity increases three times (from 0.4 to 1.2 dL g^−1^) after heating at 60 °C during 5 h. For the sample P3 the intrinsic viscosity increases by 30% after heating at 25 °C during 12 h, which is almost equivalent to the corresponding keeping at 60 °C during 2.5 h. Deviations from linearity are related to interactions between polymer coils. The obtained data suggest a real possibility of controlled annealing of PANI solutions for molecular weight increase up to the required level and achieving the required viscoelastic properties. P1 and P3 solutions upon heating at 60 °C show exponential-like molecular weight increase with the extent of conversion, since it is a condensation polymerization, data for complex viscosity change are shown in Figure S2 (Supplementary Materials).

### 3.2. Selection of a Coagulant

Four methods are available for fiber spinning from polymer solutions: wet [7,9], dry-wet jet [11], dry [15] and mechanotropic [16]. In the case of wet and dry-wet jet methods of fiber spinning, polymer isolation in a form of fibers occurs due to mass transfer processes which take place when solution jet contacts a non-solvent (coagulant). A search for coagulation conditions, particularly coagulation mixture content and temperature, as well as optimal properties of the dope allows to vary the main fiber properties such as polymer molecular orientation and its future structure, porosity, core-shell morphology, and also predetermine a fiber cross section shape [9]. All of that significantly affects final fiber morphology, structure and mechanical properties.

A solution coagulation process proceeds due to mutual coagulant diffusion into a solution jet and solvent diffusion from a jet. Process velocity is determined by concentration difference between two components, medium resistance and osmotic pressure difference which occur during the interdiffusion. The effect of main parameters which affect coagulation process could be assessed without carrying out a hard and long fiber spinning process. The assessment can be performed microscopically by interdiffusion kinetic analysis on a solution droplet which is placed between two optical glasses and surrounded by a coagulant of determined composition [7,8].

**Figure 8:**
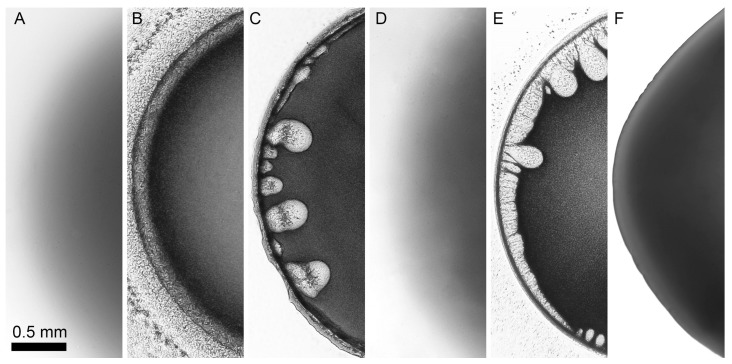
Interaction of 11% PANI P2 solution droplet in NMP with 15/85 H_2_O/NMP (**a**), 17/83 H_2_O/NMP (**b**), 20/80 H_2_O/NMP (**c**), 30/70 EtOH/NMP (**d**), 50/50 EtOH/NMP (**e**), 20/10/70 EtOH/H_2_O/NMP (**f**).

The results of P2 solution droplet coagulation process with coagulants of different content are shown in Figure 8.

Two-component coagulants that are based on NMP with water or ethanol do not provide uniform morphology evolution of a PANI droplet. Water is a strong coagulant, and the coagulation process already proceeds at more than 17% of water (Figure 8b,c). At lower water content the coagulation does not happen at all (Figure 8a). If ethanol is added to NMP, the coagulation process starts only when ethanol content exceeds 40% (Figure 8e). At ethanol content less than that the coagulation does not occur (Figure 8d). Using of ethanol-water coagulant results in many defects (vacuoles) formed in a drop. Water-ethanol-NMP mixture provides uniform coagulation of a solution droplet without defects (Figure 8f). 

**Figure 9:**
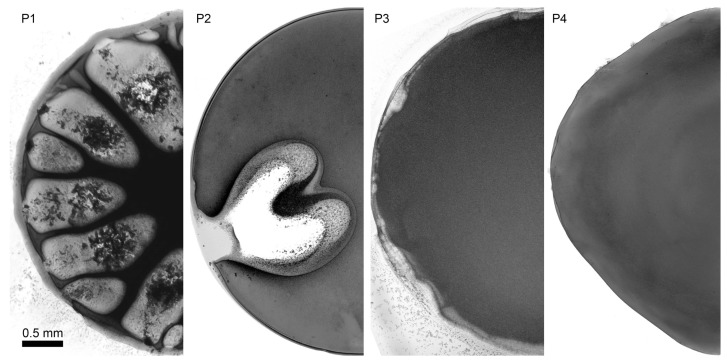
Interaction of P1-P4 solution droplet with coagulant based on mixture EtOH/H_2_O/NMP (20/10/70).

By choosing the most optimal coagulant based on ternary mixture it becomes possible to analyze the coagulation process for solutions after annealing, which leads to changes in polymer molecular weight and rheological properties. Microscopy images of P1–P4 solution droplet coagulation by a coagulant EtOH/H_2_O/NMP (20/10/70) are shown in Figure 9.

Viscoelasticity growth through P1 to P4 solutions (with preservation of the same contact angle and adhesion to glass) favors droplet shape stabilization and significant decrease of defects caused by a coagulation. For low-viscous solutions the choice of appropriate coagulant becomes extremely difficult because “soft” coagulants with high NMP content dissolve the system, and “hard” coagulants with high non-solvent content lead to defect formation similar to those shown in Figure 9 for the case of P1 and P2 coagulation. 

Therefore, the best coagulant for PANI solutions in NMP is triple mixture, which is based on ethanol/water/NMP at ratio of 20/10/70. At the same time the dopes should be viscoelastic enough for jet shape preservation during mass transfer in wet fiber spinning.

### 3.3. Fiber Spinning

**Figure 10:**
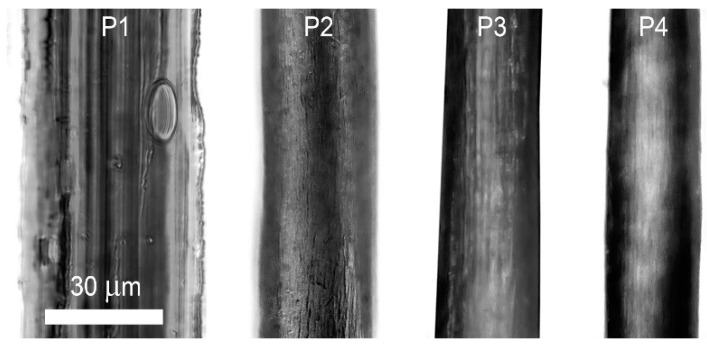
Microscopy images of fibers obtained from P1–P4 solutions.

Fibers are obtained from aforementioned solutions by the method of wet spinning with the optimal coagulant mixture. Spinnability of the solutions differs significantly. P1 solution was not spun in a stable mode. Many filament breaks were observed, which might be related to low jet extension viscosity, many defects appearing at coagulation. P2 and P3 solutions were spun more stable allowing to prepare fibers in continuous mode. During P4 solution spinning monofilament breaks occurred, likely, due to high spinbond hoods, which should be excluded. Attempts of fiber orientation stretching in air at room temperature were unsuccessful but at higher temperature this process becomes possible. Microscopy images of fibers obtained from solutions P1–P4 are shown in Figure 10.

The fibers obtained from low viscosity P1 solution are characterized by non-uniform surface which is caused by the highly strong coagulant (Figure 9). Fibers spun from P2–P4 solutions have sufficiently smoother surface and are almost defectless. It is especially related to fiber samples from the most structured P4 solution. 

Mechanical properties of the fibers are shown in Table 2. 

It can be seen that polymer molecular weight and corresponding solution viscoelasticity improve the final fiber properties. Fibers spun from P4 solution possess the highest strength and elastic modulus. Their significantly low diameter is surprising, despite the highest viscosity of P4 solution. Probably, it is related to high jet elasticity which allows high stretching. Increased value of elongation at break for P2 may be related to sufficiently different viscoelastic properties of the solutions as well as to coagulation and jet stretching. For P1 fibers low values of the elongation at break are related to the embrittlement due to high number of defects which appear during coagulation (Figure 9 and Figure 10). P2 solution, flowing from spinnerette were subjected to plastic deformation with further gel-like fiber formation during coagulation. In this case, jet stretching favored thinning of the jet as a result of a solution flow and not a polymer orientation. The obtained fibers possessed high values of relative elongation. P3 and especially P4 solutions are strongly structured. They were gelled immediately after contact with a coagulant. That is why positive jet stretching favored orientation of polymer chains in gel-like fibers. As a result, less oriented fibers with lower elongation at break were obtained.

**Figure 11:**
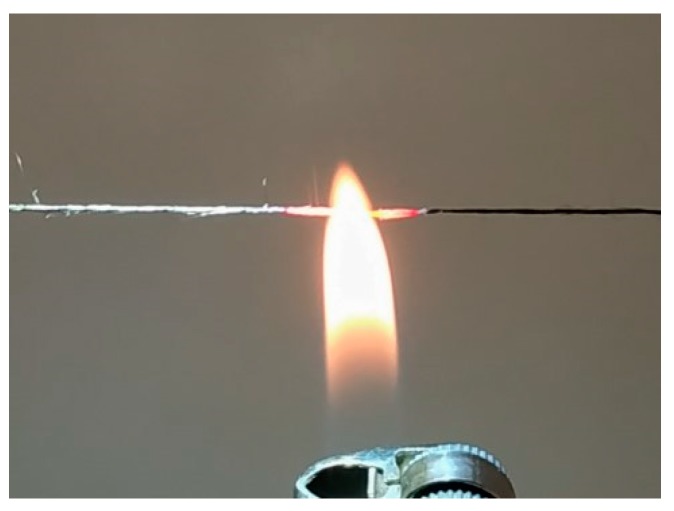
“Lola-M” fiber behavior in open flame.

It is necessary to underline thus obtained pre-polymer fibers are a subject for further treatment in order to proceed polymer cyclization and thermal stretching. That is why the thermal treatment of P4 fibers was performed at 350 °C for 4 h in the oven in argon atmosphere and flammability test was conducted. In open flame a complex fiber glows red, does not melt, does not burn and does not smolder when taken out of the flame (Figure 11).

Further investigation will be performed for PANI fiber spinnability and fiber post-treatment with “Lola-M” formation. 

## 4. Conclusions

For the first time the fibers were obtained from PANI solutions in organic solvent NMP by wet spinning using coagulating bath consisted of NMP, ethanol and water. A search for pre-polymer solution viscoelastic properties, spinning conditions including the content of coagulation bath allowed us to obtain pre-polymer fibers with diameter of ~20 µm and strength of up to 325 MPa with minimal number of defects. Mechanical properties of as-spun fibers allow their further post-treatment in order to obtain high strength incombustible “Lola-M” fibers. Preliminary test has shown that the fiber samples after annealing at 350 °C do not burn in open flame.

## Figures and Tables

**Table 1:** Properties of PANI solutions in NMP.

Properties	P1	P2	P3	P4
Concentration, % mass.	11.6	11.6	13.0	13.0
Intrinsic viscosity,* dL/g	0.4	1.2	2.8	3.8

*M_w_ can be calculated by Mark-Kuhn-Houwink equation [η] = K_η_M^α^ = 1.1·10^−4^ M_w_^0.89^ [6].

**Table 2:** Mechanical properties of fibers obtained from P1-P4 solutions without thermal treatment.

Sample	Strength, MPa	Elongation at break, %	Modulus of elasticity, GPa	Diameter, µm
**P1**	32 ± 40	10 ± 20	0.6 ± 0.3	34 ± 5
**P2**	132 ± 15	47 ± 10	1.3 ± 0.2	24 ± 3
**P3**	144 ± 20	19 ± 7	1.5 ± 0.1	23 ± 4
**P4**	325 ± 50	19 ± 6	2.8 ± 0.4	19 ± 5

## Supplementary Materials

The following are available online at https://www.mdpi.com/1996-1944/12/21/3490/s1, Figure S1: G’ and G’’ data for P3 and P4 solutions (a) 25 °C, (b) 60 °C, Figure S2: Exponential increase of viscosity, Table S1: Flow time for P3.
