# Supplementary material for: New Approach to Preparation of Heat-Resistant “Lola-M” Fiber
Source: Materials (Basel). 2019 Oct 25;12(21):3490. doi: 10.3390/ma12213490 (PMC6862234; doi:10.3390/ma12213490)
Supplement: Supplementary file 1 [file materials-12-03490-s001.pdf]

materials

Supplementary Materials

New Approach to Preparation of Heat-Resistant "Lola-M" Fiber

Igor I. Ponomarev [1,*], Ivan Y. Skvortsov [2], Yulia A. Volkova [1], Ivan I. Ponomarev [1], Lydia A. Varfolomeeva [2], Dmitry Y. Razorenov [1], Kirill M. Skupov [1,*], Mikhail S. Kuzin [2] and Olga A. Serenko [1]

1 A.N. Nesmeyanov Institute of Organoelement Compounds of Russian Academy of Sciences, Vavilova St., 28, Moscow, 119991, Russia

2 A.V. Topchiev Institute of Petrochemical Synthesis of Russian Academy of Sciences, Leninsky Av., 29, Moscow, 119991, Russia

* Correspondence: gagapon@ineos.ac.ru (I.I.P.); kskupov@gmail.com (K.S.)

1. G′ and G″ Data for P3 and P4 Solutions

G′ and G″ data for P3 solution heated at 60 °C, 2.5 h, compared to fresh solution at 60 °C and G′ and G″ data for P3 solution at 25 °C compared with P3 solution after 25 °C, 12 h (which is actually P4 solution) are shown below.

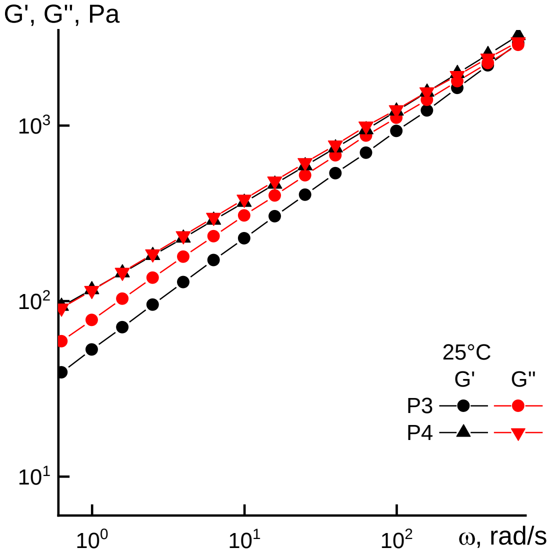

(**a**)

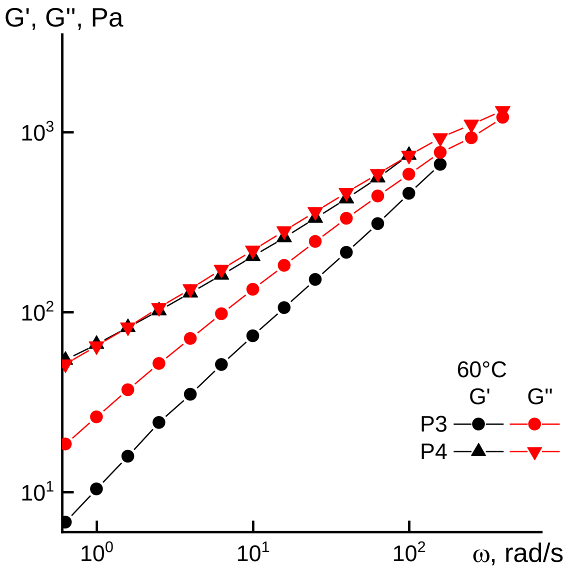

(**b**)

Figure S1. G′ and G′′ data for P3 and P4 solutions (**a**) 25 °C, (**b**) 60 °C

2. Flow Time for P3

Flow times (Ubbelohde viscometer) for P3 solution for different concentrations are shown below in the table.

Table S1. Flow time for P3

c, g/mL	t, s
0	123
0.13	175
0.3	258
0.51	385
1.04	937

3. Exponential Increase of Viscosity

Both solutions, P1 and P3, upon heating at 60°C show exponential-like molecular weight increase with the extent of conversion, since this is a condensation polymerization. Data for complex viscosity change are shown below.

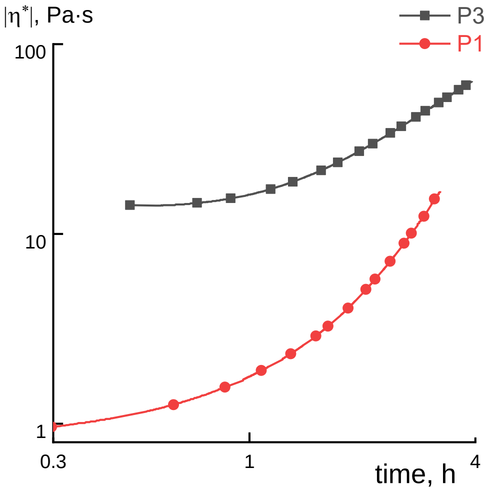

Figure S2. Exponential increase of viscosity

© 2019 by the authors. Submitted for possible open access publication under the terms and conditions of the Creative Commons Attribution (CC BY) license (http://creativecommons.org/licenses/by/4.0/).
